# Antiliver Fibrosis Screening of Active Ingredients from *Apium graveolens* L. Seeds via GC-TOF-MS and UHPLC-MS/MS

**DOI:** 10.1155/2020/8321732

**Published:** 2020-02-18

**Authors:** Ming Qiao, Jianhua Yang, Yao Zhao, Yi Zhu, Xiaomei Wang, Xinling Wang, Junping Hu

**Affiliations:** ^1^College of Pharmacy, Xinjiang Medical University, Urumqi 830011, China; ^2^Department of Pharmacy, The First Affiliated Hospital, Xinjiang Medical University, Urumqi 830011, China

## Abstract

Although several studies have been performed on *Apium graveolens* L.(celery) seeds, their antiliver fibrosis effects remain to be unexplored. Firstly, we detected the effects of celery seeds extracted with different concentrations of aqueous ethanol on the proliferation of HSC-LX2 cells. Then, we detected the effects of fractions of the optimal effect extract on the proliferation and apoptosis of HSC-LX2 cells. Finally, the compounds of petroleum ether (PP), ethyl acetate (PE), n-butyl alcohol (PB), and water fractions (PW) of the optimal effect extract were determined by GC-TOF-MS and UHPLC-MS/MS, to confirm the potentially antifibrotic compounds combined with pharmacodynamic experiment of monomer compounds *in vitro*. The results revealed that 60% ethanol extract of celery seeds (60-extract) exhibited remarkable inhibition effect on the proliferation of HSC-LX2 cells compared with 95% ethanol and aqueous extract. Besides, it validated that the inhibition rates of PP, PE, PB, and PW on the proliferation of HSC-LX2 cells were 75.14%, 73.52%, 54.09%, and 43.36%, and their percentage of apoptotic cells were 37.5%, 4.3%, 0.7%, and 0.1% at high doses, respectively. Additionally, it was manifested that apigenin, aesculetin, and butylphthalide have major contribution to the overall compounds of celery seeds, and the inhibition effects on the cell proliferation clearly elevated with increase in their contents. In essence, apigenin, aesculetin, and butylphthalide may hopefully become the natural products of antiliver fibrosis, which laid a foundation for the subsequent development of celery seeds as antiliver fibrosis drugs.

## 1. Introduction

Liver fibrosis is the essential pathophysiologic consequence of chronic hepatic injury [1]. Without favorable treatment, liver fibrosis can develop into cirrhosis, which is estimated to affect 1% to 2% of global population and result in over 1 million deaths annually worldwide [2, 3]. The major causes of liver fibrosis include chronic hepatitis virus infection, alcohol abuse, and nonalcoholic steatohepatitis [4]. Hepatic fibrosis is characterized by excessive accumulation of extracellular matrix (ECM) caused by both increased synthesis and deposition of newly formed components and decreased or unbalanced degradation of ECM [5]. Hepatic stellate cell (HSC) activation is considered as a pivotal event in liver fibrosis, which is mainly responsible for the excessive accumulation of ECM proteins in the liver [6, 7]. Nowadays, a growing body of basic and clinical evidences have manifested that liver fibrosis can be reversed after the cessation of injurious stimulus [8, 9]. Hence, the development of antiliver fibrosis drugs is an urgent demand at present.

*Apium graveolens* L.(celery) belongs to the family Apiaceae, which is grown as a vegetable in many parts of the world [10]. It is rich in compounds including limonene, selinene, glycosides, flavonoids, and vitamins [11]. These constituents are also reported to prevent cardiovascular diseases [12], jaundice [13], urinary tract obstruction [14], gout [15], and rheumatic disorders [16, 17]. Celery seeds are spicy, carminative [18], diuretic [19], appetizer [20], stimulant, hypotensive [21], aphrodisiac, anti-inflammatory, and laxative [22]. In addition, celery seeds are also widely used in Chinese compound preparations to protect the liver, such as Huganbuzure Granule, Ganbaokang Granule, Fufangzupa Syrup, and Mawuliwusuli Granule [23, 24]. However, there is no report for pharmacological activity of celery seeds on the antiliver fibrosis. Therefore, transforming growth factor-*β*1 (TGF-*β*1) induced HSC-LX2 cells to establish a liver fibrosis model *in vitro* to investigate the antiliver fibrosis effect of active fractions and compounds of celery seeds combined with GC-TOF-MS and UHPLC-MS/MS techniques.

## 2. Materials and Methods

### 2.1. Materials

*Apium graveolens* L. seeds were purchased from the decoction factory of Xinjiang Madison Medicine (Xinjiang, China). The botanical identification of the plant material was performed by Dr. Jianhua Yang from the first affiliated hospital of Xinjiang Medical University. The voucher specimen was deposited at the department of pharmacognosy of Xinjiang Medical University. Methanol, acetonitrile, and formic acid (HPLC grade) were obtained from Merck (Darmstadt, Germany). L-2-chlorophenylalanine and BSTFA (HPLC grade) were supplied by Sigma (St. Louis, MO, USA). Ultrapure water was prepared with a Milli-Q system (Millipore, Milford, MA, USA). Ethyl alcohol, petroleum ether, ethyl acetate, and n-butyl alcohol were of analytical grade.

#### 2.1.1. Preparation of the Extracts

The celery seed extracts were prepared according to the reflux extraction, by adding 8 times the amount of distilled water, 60% and 95% aqueous ethanol (v/v) to 50.00 g of the dried celery seeds, respectively, and heating reflux 3 times (3 × 1.5 h) at 60°C. The filtrates were combined and concentrated with a rotary evaporator (IKA, Staufen, Germany) to obtain aqueous extract, 60% and 95% ethanol extracts, and the yields were 8.6%, 3.8% and 12.8%, respectively.

#### 2.1.2. Preparation of the Fractions and Monomeric Compounds from Celery Seeds

Celery seeds (8.00 kg) were refluxed thrice with 8 times the amount of 60% ethanol at 60°C, and the extracts were combined and evaporated under reduced pressure to afford 200.6 g of crude extract. The 60% ethanol extract was dissolved in water to produce an aqueous solution, and then partitioned in turn with petroleum ether, ethyl acetate, and n-butyl alcohol to afford petroleum ether (PP, 5.12 g), ethyl acetate (PE, 20.80 g), n-butyl alcohol (PB, 70.60 g), and water-soluble (PW, 89.60 g) fractions after drying. Nine compounds were isolated from PP, PE, PB, and PW. Their structures were identified as apigenin (8.3 mg) [25], 5-methoxypsoralen (10.2 mg) [26], apiin (9.1 mg) [27], rutin (7.7 mg) [28], kaempferol (9.3 mg) [29], luteolin (6.1 mg) [30], quercetin (6.2 mg) [31], mollugin (5.6 mg) [32], and butylphthalide (8.0 mg) [33] by using extensive spectroscopic analysis including ^1^H-NMR and ^13^C- NMR.

#### 2.1.3. Samples for GC-TOF-MS and UHPLC-MS/MS Analysis

100 *μ*L of PP was extracted with methanol and added 10 *μ*L of L-2-chlorophenylalanine as internal standard and vortex (Grant, Cambridge, UK) mixed for 30 seconds. Then it was centrifuged at 4°C for 15 min at 12000 rpm using a centrifuge (Thermo Fisher, Waltham, USA). 100 *μ*L of supernatant was transfered and dried completely in a vacuum concentrator (Retsch, Arzberg, Germany). To add 60 *μ*L of the BSTFA reagent to the sample aliquots, it is incubated for 1.5 hours at 70°C. The sample was analysed by GC-TOF-MS. The PE, PB, and PW were thawed on the ice. Then the samples were centrifuged at 12000 rpm for 15 min at 4°C, and 200 *μ*L of supernatant of the sample was dried under a gentle nitrogen flow. The residue was reconstituted with 200 *μ*L of methanol. Then the samples were centrifuged at 12000 rpm for 10 minutes at 4°C. The resulting supernatants were transferred to 2 mL of sample vials and stored at −80°C until the UHPLC-MS/MS analysis.

### 2.2. Cell Culture

HSC-LX2 cells (Applied Biosystems, Foster City, USA) were cultured in a high-glucose Dulbecco's modifed eagle medium (HG-DMEM, Gibco, Grand Island, USA) supplemented with 10% FBS (Gibco, Grand Island, USA), 100 *μ*g·mL^−1^ streptomycin, and 100 U·mL^−1^ penicillin (Gibco, Grand Island, USA). The cells were maintained in a humidified incubator with 5% CO_2_ at 37°C, and they were subcultured every 2 days to maintain logarithmic growth.

### 2.3. Establishment of the Liver Fibrosis Model Induced by TGF-*β*1

The logarithmic phase cells were inoculated in a 96-well plate at a density of 2 × 10^3^ cells per well and treated with TGF-*β*1 (Novoprotein, Shanghai, China) at concentrations of 5, 10, 20, 40, 80, and 100 ng·mL^−1^ for 24, 48, and 72 h. Blank group (containing all reagents except the studied sample) was set. The cell viability was assessed by cell-counting kit-8 (CCK-8) assay (Solarbio, Beijing, China). The absorbance was determined at 450 nm using multiscan spectrum (Multiskan GO, Thermo Fisher, Waltham, USA). The hyaluronic acid (HA), laminin (LN), and type III procollagen (PCIII) levels were determined using enzyme-linked immunosorbent assay (ELISA) kits (Jianglai Biological, Shanghai, China). All conditions were performed in triplicate, and each experiment repeated for three times. Cell viability was calculated as follows:

(1) $$\begin{array}{c} \text{Cell viability}\left(\%\right)=\left[\frac{\left({\text{OD}}_{450\left(\text{TGF}-\beta 1\right)}-{\text{OD}}_{450\left(\text{solvent}\right)}\right)}{\left({\text{OD}}_{450\left(\text{blank}\right)}-{\text{OD}}_{450\left(\text{solvent}\right)}\right)}\right]\times 100. \end{array}$$

### 2.4. Cell Proliferation Assay

HSC-LX2 cells were plated on 96-well plates at a density of 5 × 10^3^ cells per well and treated with various concentrations of extracts and fractions of celery seed in combination with 100 ng·mL^−1^ of TGF-*β*1 for 48 h. Blank group (containing all reagents except the studied sample) was set. The positive control group was added with 50 *μ*g·mL^−1^ of the compound Biejiaruangan Troche (BJRG) (Inner Mongolia Furui Medical Science, Inner Mongolia, China). 100 ng·mL^−1^ of TGF-*β*1 was added to the model group. The absorbance was quantified at 450 nm using a multiscan spectrum. Additionally, the HA, LN, and PCIII levels were also performed by ELISA kits. Each treatment was performed in triplicate, and each experiment was repeated for three times. Cell inhibiting rate was calculated as follows:

(2) $$\begin{array}{c} \text{cell inhibiting rate }\left(\%\right)=\left[1-\left[\frac{\left({\text{OD}}_{450\left(\text{sample}\right)}-{\text{OD}}_{450\left(\text{solvent}\right)}\right)}{\left({\text{OD}}_{450\left(TGF-\beta 1\right)}-{\text{OD}}_{450\left(\text{solvent}\right)}\right)}\right]\right]\times 100. \end{array}$$

### 2.5. Cell Apoptosis

Annexin V-FITC Apoptosis Detection Kit (Becton Dickinson, Franklin Lakes, NJ, USA) was utilized for cell apoptosis analysis according to the manufacturer's instruction. Cells were divided into viable cells, dead cells, early apoptotic cells and apoptotic cells, and analyses were performed on the flow cytometer (Becton Dickinson, Franklin Lakes, NJ, USA). The ratio of apoptotic cell reflected the proapoptotic effect of PP, PE, PB, and PW of 60% ethanol extract from celery seeds. Each treatment was repeated in triplicate.

### 2.6. GC-TOF-MS Analysis

The Agilent 7890 gas chromatograph system is coupled with a Pegasus HT time-of-flight mass spectrometer. The system utilized a DB-5MS capillary column coated with 5% diphenyl cross-linked with 95% dimethylpolysiloxane (30 m × 250 *μ*m, 0.25 *μ*m; J&W Scientific, Folsom, CA, USA). An aliquot of the analyte (1 *μ*L) was injected into the GC/MS apparatus. Helium was the carrier gas at a flow rate of 1 mL·min^−1^. GC oven temperature started at 50°C and was held for 1 min at 310°C and then for 10 min with program rate 10°C·min^−1^. The injector and detector temperatures were set at 280°C and 250°C, respectively. The mass spectrometer was run in the electron ionization mode (−70 eV).

### 2.7. UHPLC-MS/MS Analysis

Chromatographic analysis was performed in an Agilent 1290 ultra-high performance liquid chromatography (Agilent, Technologies, CA, USA). An ACQUITY ULC HSS T3 column (2.1 mm × 100 mm, 1.8 *μ*m, Waters, USA) was used for separation with gradient elution of 0.1% formic acid aqueous solution (A) and acetonitrile (B), and the column temperature was maintained at 40°C. The detailed gradient conditions were 0–2 minutes, 0%–2% B; 2–11 minutes, 2%–98% B; and 11–13 minutes, 98%–2% B. The flow rate was 0.3 mL·min^−1^, and the injection volume was 10 *μ*L. Mass spectrometry was performed on the AB Sciex QTrap 6500 (AB SCIEX, Los Angeles, CA, USA) for characterization equipped with electrospray ionization (ESI) source in both positive and negative ionization multiple reaction monitoring (MRM) mode. The parameters were set as follows: nebulizer gas of 60 psi; heater gas of 55 psi; curtain gas of 35 psi; ionspray voltage of +5500/−4500 V; ion transfer tube temperature of 550°C.

### 2.8. Screening of Antiliver Fibrosis Activity of Monomeric Compounds

Briefly, cells were seeded onto a 96-well plate at a density of 5 × 10^3^ cells per well. Cells were exposed to different concentrations of compounds with 100 ng·mL^−1^ TGF-*β*1 24 h after seeding. The positive control group was added with 50 *μ*g·mL^−1^ of BJRG. Each treatment was repeated in triplicate. After 48 h of sample incubation at 37°C, CCK-8 solution was added to each well. After a further 1 h of incubation, the absorbance of each well was measured at 450 nm, and cell inhibiting rate was calculated.

### 2.9. Statistical Analysis

The results of cell experiment were expressed as the mean standard deviation of three parallel measurements, and statistical significance was assessed using one-way analysis of variance (ANOVA) followed by a multiple comparison test (Tukey's post-hoc test), where *P* < 0.05 and *P* < 0.01 were considered significant. Three injections used in the GC-TOF-MS and UHPLC-MS/MS analysis, and the relative percentage of the compound was the average of the normalized values of the three determinations of the peak area.

## 3. Results

### 3.1. Effects of TGF-*β*1 on the Proliferation of HSC-LX2 Cells

We used six concentrations and three time points of TGF-*β*1 to explore the proliferation of HSC-LX2 cells (Table 1). At given time points, HSC-LX2 cells viability clearly elevated with increased TGF-*β*1 concentrations. The HSC-LX2 cells viability remarkably decreased with prolonged time. Absorbance peaked at 48 h with 100 ng·mL^−1^ of TGF-*β*1 and abated gradually later. Therefore, HSC-LX2 cells were activated by 100 ng·mL^−1^ of TGF-*β*1 for 48 h to establish a liver fibrosis model.

### 3.2. Effects of TGF-*β*1 on Liver Fibrosis Indexes

As expected, serum HA, LN, and PCIII levels were remarkably elevated after TGF-*β*1 treatment in comparison with the blank control group. It indicated that the liver fibrosis model was established successfully *in vitro* (Table 2).

### 3.3. Effects of Extracts and the Fractions of Celery Seeds on Proliferation of HSC-LX2 Cells

HSC-LX2 cells were notably activated by TGF-*β*1, as shown in Table 3. The results indicated that celery seed extracts and its fractions inhibited HSC-LX2 cell proliferation in a dose-dependent fashion. 60% ethanol extract showed a stronger inhibitory effect than the aqueous extract and 95% ethanol extract. The inhibition rates of PP, PE, PB, and PW were 75.14%, 73.52%, 54.09%, and 43.36% at high doses, respectively.

### 3.4. Effects of PP, PE, PB, and PW on Liver Fibrosis Indexes

TGF-*β*1 treatment caused a remarkable collagen accumulation. In sharp contrast, treatment with PW, PB, PE, and PP markedly decreased hepatic collagen matrix accumulation, as shown in Table 4. The serum LN, HA, and PCIII contents were markedly reduced in the PE and PP groups, and that of PP groups were closer to the control groups. PW, PB, PE, and PP exhibited different degree in antiliver fibrosis, which might be related to the active component contents.

### 3.5. Apoptosis Analysis

**Figure 1:**
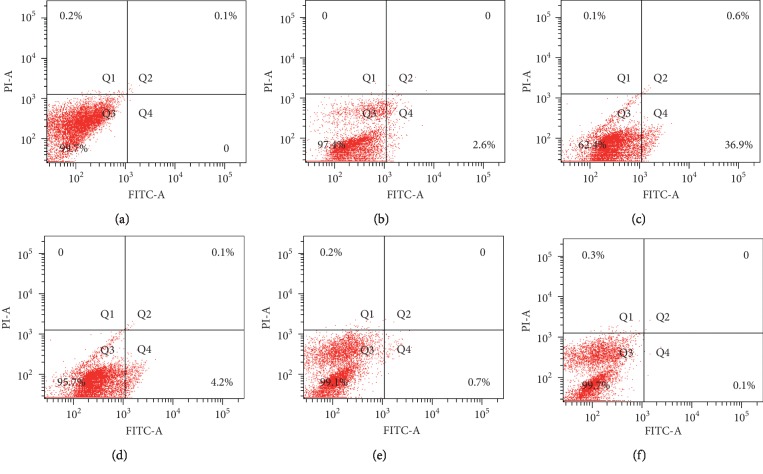
Effects of PP, PE, PB, and PW on apoptosis of HSC-LX2 cells. (a) Control. (b) BJRG. (c) PP. (d) PE. (e) PB. (f) PW.

Annexin V-FITC/PI double staining and flow cytometry were performed to compare the apoptotic rates of HSC-LX2 cells in different groups. As shown in Figure 1, the percentage of apoptotic cells was 0.1% in the control group (Figure 1(a)), that of the group treated with BJRG, PP, PE, PB, and PW were 2.6%, 37.5%, 4.3%, 0.7%, and 0.1%, respectively (Figures 1(b)–1(f)). PP showed stronger proapoptotic function than that of the positive control.

### 3.6. Analysis of the Fractions of Celery Seeds Using GC-TOF-MS and UHPLC-MS/MS Methods

**Figure 2:**
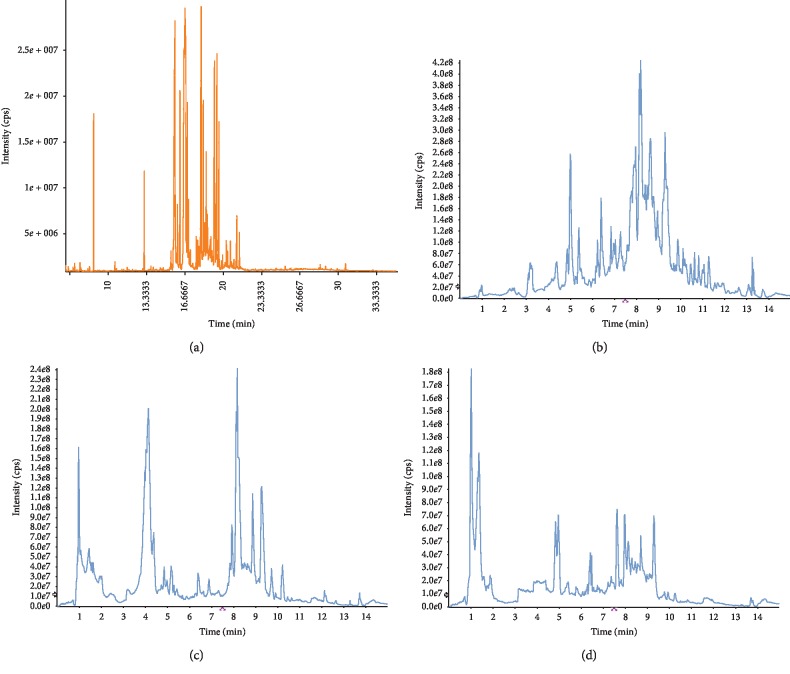
Total ion chromatograms of fractions of celery seeds. (a) PP, (b) PE, (c) PB, and (d) PW.

Identification of several compounds was enabled by comparison of retention times, MS spectra, and MS/MS spectral data with the standard substance and reference materials reported in the literature. According to our knowledge, the chemical compositions of the fractions from celery seeds, investigated by GC-TOF-MS and UHPLC-MS/MS, was performed for the first time in this study. 122 chemical components were identified from PP, PE, PB, and PW through analysis of GC-TOF-MS and UHPLC-MS/MS (Table 5). The obtained total ion chromatograms are shown in Figure 2. The 378 peaks of PP were separated in GC and preliminarily identified 39 main compounds belonging to different chemical families by TOF-MS which account for 94.32% of total peak area. Chemical composition analysis showed that phenyl peptides accounted for 24.56% acids, 22.96% aldehydes 19.37%, flavonoids 18.42%, alcohols 3.24%, and major compounds were farnesal 19.37%, butylphthalide 18.42%, 4-hydroxymethyl 3-methoxyphenoxyacetic acid 13.54%, aesculetin 13.28%, and apigenin 11.28%.

Of all the compounds detected by UHPLC-MS/MS, 90 compounds were identified by searching the Biotree DB Database and comparing their retention times and MS spectra with the reference literature. 52 chemical constituents were identified from PE, mainly flavonoids, amides, pigments, and ketones, accounting for 75.86% of the total peak area. The major constituents that have been determined through UHPLC-MS/MS analyses were apigenin 8.48%, 2-acetyl-1-ethylpyrrole 5.22%, aesculetin 4.32%, and 2-phenylacetamide 3.86%. 48 compounds were identified from PB, mainly glycosides, esters, aldehydes, and ketones, accounting for 81.88% of the total peak area. The main chemical constituents were adenosine 14.99%, 15-methylpalmitate 7.18%, ambolin 6.72%, 2-hydroxymuconate semialdehyde 5.21%, and chalconaringenin 4.87%. 58 chemical compositions were identified from PW, mainly sugars, glycosides, acids, and salts, accounting for 84.35% of the total peak area. Nipecotic acid 11.99%, 3-methyl 1-phenyl-3-pentanol 8.05%, mesylate 7.13%, and luteolin 7-galactoside 6.59% were the main chemical constituents.

Aesculetin, 2-phenylacetamide, 1H-indole-3-carboxaldehyde, alpha-terpinene, and erucamide all existed in PP, PE, PB, and PW, and aesculetin accounted for 13.28%, 4.32%, 0.56%, and 0.11%. PB, and PW had 19 components in common, including 2-phenylacetamide, 2-acetyl-1-ethylpyrrole, apiin, 2-hydroxymuconate semialdehyde, and 15-methylpalmitate. Apigenin was found in both PP and PE, accounting for 11.28% and 8.48%. It was suggested that the PW, PB, PE and PP exhibited different degree in antiliver fibrosis, which might be related to the active compounds contents combined with the pharmacodynamic experiments and the analysis of components.

### 3.7. Potential Antiliver Fibrosis Activity of Monomeric Compounds

All apigenin, aesculetin, butylphthalide, quercetin, apiin, kaempferol, and rutin had inhibition effects on HSC-LX2 cell proliferation, and the inhibition effect enhanced with increased compound concentrations (Table 6). Their inhibition rates of high-dose were 71.32%, 64.90%, 61.93%, 48.28%, 30.87%, 22.02%, and 15.81%, respectively. 5-methoxypsoralen, luteolin, and mollugin did not have inhibition effects on the proliferation of HSC-LX2 cells. The above results confirmed the antiliver fibrosis compositions of celery seeds and clarified that differences of antiliver fibrosis effects among PW, PB, PE, and PP.

## 4. Discussion

Liver fibrosis is a critical link in the development of cirrhosis or hepatocellular carcinoma, and there is presently no effective treatment for liver fibrosis. Consequently, it is indispensable to develop new drugs to ameliorate liver fibrosis [34]. In the preceding work, it presented that the alcohol extract of celery seeds could alleviate liver fibrosis. The outcomes of the current work were in keeping with those of earlier studies and further revealed that apigenin, aesculetin, and butylphthalide might have major contribution to the overall antiliver fibrosis activity of celery seeds. In short, it implied that celery seeds might possess potential treatment effect in liver fibrosis.

Transforming growth factor-*β*1 (TGF-*β*1) was considered a vital role in the process of liver fibrosis, which could affect hepatic stellate cell (HSC) activation and collagen deposition [35–38]. Therefore, TGF-*β*1 is widely employed to establish a liver fibrosis model *in vitro*. The activated HSCs lead to hepatic fibrogenesis by excessive production of extracellular matrix (ECM) components [39], such as hyaluronic acid (HA), laminin (LN), and type III procollagen (PCIII) [40–42]. Liver fibrosis is associated with major alterations in both quantity and composition of ECM [43]. In advanced stage, fibrotic liver contains three to ten times more ECM than the normal liver [44]. Estimations of serum HA, LN, and PCIII have good prognostic value for liver fibrosis complications [45, 46]. Compound Biejiaruangan Troche (BJRG) was an SFDA-approved antifibrotic medicine, which was selected as the positive control in this work [47]. It could achieve the effect of preventing and treating liver fibrosis in multiple links and targets. In this work, 60% ethanol extract of celery seeds (60-extract) showed higher inhibitory rate of HSC-LX2 cells than that of aqueous extract and 95% ethanol extract. The results of follow-up research manifested that inhibitory effects of petroleum ether (PP) and ethyl acetate (PE) fractions of 60-extract on the proliferation of HSC-LX2 cells stronger than that of n-butyl alcohol (PB) and water-soluble (PW) fractions. Moreover, PP could significantly decrease the contents of HA, LN, and PCIII, which were closer to the positive control. Additionally, PP showed a remarkable proapoptotic effect of HSC-LX2 cells compared with PW, PB, and PE. It concluded that PP could effectively inhibit proliferation and promote apoptosis of HSC-LX2 cells, thereby inhibiting the ECM deposition. Mechanistically, screening of antiliver fibrosis activity of monomeric compounds exhibited that the inhibition rates at high dose of apigenin, aesculetin, butylphthalide, quercetin, apiin, kaempferol, and rutin were 71.32%, 64.90%, 61.93%, 48.28%, 30.87%, 22.02%, and 15.81%, respectively. It certified that apigenin, aesculetin, and butylphthalide have major contribution to the antiliver fibrosis activity of celery seeds.

The powerful antifibrotic activity of fractions in celery seeds may be explained by its richness in apigenin, aesculetin, and butylphthalide. This result was consistent with the study by Lee et al. [48], which proved that ethanol-induced cytotoxicity in HepG2 cells and mice were obviously prevented after treatment with aesculetin by the upregulation of antioxidant defense enzymes in the Nrf2/ARE pathway of hepatocytes. Anuradha et al. [49] confirmed the ability of aesculetin to attenuate hepatic fibrosis in NAFLD and its effect on FoxO1 activity. Wang et al. [50] explained that apigenin might exert a protective effect on alcohol-induced liver injury, which might be related to the regulations of hepatic CYP2E1-mediated oxidative stress and PPAR*α*-mediated lipogenic gene expression. It was also discovered that hepatic steatosis and inflammation were ameliorated with treatment of apigenin via regulation of the XO/NLRP3 pathways [51]. Additionally, butylphthalide has been reported in the treatment of cerebrovascular disease. Qiu et al. [52] proved that activation of microglia was prevented; meanwhile, dopaminergic neurons in the substantia nigra were preserved by butylphthalide. Nevertheless, this work first found that butylphthalide has antiliver fibrosis activity in vitro, which provides a theoretical basis for further research on it.

In summary, this work comprehensively and systematically identified the active constituents of celery seeds by GC-TOF-MS and UHPLC-MS/MS and enriched the chemical constituents of celery seeds at this stage. It illustrated that antiliver fibrosis activity clearly elevated with these increased active composition contents. On the whole, a reliable and rapid strategy was successfully established for screening of the antiliver fibrosis activity and active ingredients in celery seeds. PP was verified to be the active fraction of celery seeds with the most powerful antiliver fibrosis effect, and apigenin, aesculetin, and butylphthalide might well be the potential ingredients of antiliver fibrosis activity in celery seeds. To sum up, it is of great significance to elucidate the material basis and pharmacodynamic components of Huganbuzure Granule, Ganbaokang Granule, Fufangzupa Syrup, and Mawuliwusuli Granule of Chinese compound preparations. In addition, apigenin, aesculetin, and butylphthalide may hopefully become the natural products of antiliver fibrosis, which laid a foundation for the subsequent development of celery seeds as antiliver fibrosis drugs.

## 5. Conclusions

According to the biological and chemical analysis of celery seeds, the petroleum ether fraction was verified to be the active fraction of celery seeds with the most powerful antiliver fibrosis effect, and apigenin, aesculetin, and butylphthalide might well be the potential ingredients of antiliver fibrosis activity in celery seeds.

## Figures and Tables

**Table 1:** Effects of TGF-*β*1 on the viability of HSC-LX2 cells (mean ± SD, *n* = 3).

Group	Dose (ng·mL^−1^)	24 h		48 h		72 h	
		OD	Viability (%)	OD	Viability (%)	OD	Viability (%)
Solvent control	—	0.183 ± 0.004	—	0.175 ± 0.004	—	0.210 ± 0.001	—

Blank control	—	0.395 ± 0.102	—	0.671 ± 0.023	—	1.739 ± 0.197	—

TGF-*β*1	5	0.347 ± 0.054	77.35 ± 0.75	0.711 ± 0.072	108.11 ± 3.26	0.573 ± 0.109^#^	23.76 ± 2.65
	10	0.501 ± 0.028	150.72 ± 0.62	0.775 ± 0.042	121.10 ± 2.61	0.596 ± 0.035^#^	25.24 ± 2.41
	20	0.511 ± 0.067	154.71 ± 0.86	0.820 ± 0.103	130.12 ± 4.51	1.316 ± 0.136	47.63 ± 1.04
	40	0.695 ± 0.105^#^	215.52 ± 1.96	0.894 ± 0.110	145.21 ± 5.62	1.311 ± 0.185	72.13 ± 3.65
	80	0.640 ± 0.108^#^	241.49 ± 0.21	1.093 ± 0.079^#^	177.59 ± 2.54	2.472 ± 0.145^#^	148.51 ± 2.54
	100	0.803 ± 0.101^#^	292.45 ± 0.26	1.755 ± 0.081^##^	318.39 ± 6.26	2.728 ± 0.177^#^	164.54 ± 3.22

Compared with the blank control group (^#^*P* < 0.05, ^##^*P* < 0.01).

**Table 2:** Effects of TGF-*β*1 on liver fibrosis indexes (mean ± SD, *n* = 3).

Group	Dose (ng·mL^−1^)	LN (*μ*g·L^−1^)	HA (ng·L^−1^)	PCIII (*μ*g·L^−1^)
Blank control	—	51.8 ± 0.78	26.9 ± 5.67	18.8 ± 4.53
Model group (TGF-*β*1)	100	833.4 ± 9.94^##^	302.3 ± 7.44^##^	115.3 ± 3.38^##^

Compared with the blank control group (^#^*P* < 0.05, ^##^*P* < 0.01).

**Table 3:** Effects of the extracts and fractions of celery seeds on inhibition of HSC-LX2 cell proliferation (mean ± SD, *n* = 3).

Group	Fraction	Dose (*μ*g·mL^−1^)	OD	Inhibition ratio (%)
Solvent control		—	0.254 ± 0.019	—
Blank control		0	1.021 ± 0.079	—
Model group (TGF-*β*1)		0.1	3.522 ± 0.178^##^	—
BJRG		50	1.116 ± 0.012^*∗∗*^	73.11 ± 0.87

Aqueous extract of celery seeds		50	3.532 ± 0.227^##^	—
		100	3.074 ± 0.131^*∗*##^	13.79 ± 2.91
		200	2.537 ± 0.541^*∗*#^	30.13 ± 1.02

95% ethanol extract of celery seeds		50	3.602 ± 0.281	—
		100	2.926 ± 0.0526^*∗*#^	18.22 ± 1.69
		200	1.717 ± 0.026^*∗∗*#^	55.22 ± 2.44

60% ethanol extract of celery seeds		50	2.123 ± 0.138^*∗∗*#^	42.75 ± 2.21
		100	1.351 ± 0.052^*∗∗*^	66.42 ± 1.53
		200	1.097 ± 0.022^*∗∗*^	74.20 ± 2.38
	PP	25	2.598 ± 0.224^*∗*#^	28.26 ± 1.29
		50	1.619 ± 0.129^*∗∗*#^	58.21 ± 2.11
		75	1.066 ± 0.023^*∗∗*^	75.14 ± 1.45
	PE	25	2.893 ± 0.159^*∗*#^	19.23 ± 1.32
		50	2.001 ± 0.239^*∗∗*#^	46.55 ± 3.87
		75	1.119 ± 0.312^*∗∗*^	73.52 ± 2.36
	PB	25	2.956 ± 0.239^*∗*#^	17.30 ± 3.13
		50	1.916 ± 0.209^*∗∗*#^	49.24 ± 2.03
		75	1.754 ± 0.241^*∗∗*#^	54.09 ± 1.44
	PW	25	3.369 ± 0.319^##^	4.68 ± 0.75
		50	2.889 ± 0.162^*∗*#^	19.36 ± 1.72
		75	2.105 ± 0.311^*∗∗*#^	43.36 ± 3.55

Compared with the blank control group (^#^*P* < 0.05, ^##^*P* < 0.01); compared with the model group (^*∗*^*P* < 0.05, ^*∗∗*^*P* < 0.01).

**Table 4:** Effects of PP, PE, PB, and PW on liver fibrosis indexes (mean ± SD, *n* = 3).

Group	Dose (*μ*g·mL^−1^)	LN (*μ*g·L^−1^)	HA (ng·L^−1^)	PCIII (*μ*g·L^−1^)
Blank control	0	51.8 ± 0.78	26.9 ± 5.67	18.8 ± 4.53
BJRG	50	211.0 ± 5.08^*∗∗*^	64.5 ± 3.19^*∗*^	29.2 ± 2.53^*∗∗*^
Model group (TGF-*β*1)	0.1	833.4 ± 9.94^##^	302.3 ± 7.44^##^	115.3 ± 3.38^##^
PW	75	638.27 ± 7.22^#ΔΔ^	178.82 ± 3.94^#*∗*Δ^	108.87 ± 9.29^##ΔΔ^
PB	75	502.56 ± 5.85^#*∗*ΔΔ^	111.37 ± 4.95^#*∗*Δ^	78.36 ± 5.22^#Δ^
PE	75	118.40 ± 5.74^*∗∗*^	70.80 ± 7.07^*∗*^	28.30 ± 0.21^*∗∗*^
PP	75	97.32 ± 6.30^*∗∗*^	30.91 ± 5.22^*∗∗*^	19.01 ± 4.26^*∗∗*^

Compared with the blank control group (^#^*P* < 0.05, ^##^*P* < 0.01); compared with the model group (^*∗*^*P* < 0.05, ^*∗∗*^*P* < 0.01); compared with the PP group, (^Δ^*P* < 0.05, ^ΔΔ^*P* < 0.01).

**Table 5:** Identification of chemical compositions of fractions from celery seeds.

No.	RT^a^ (min)	Compound	Peak Area^b^ (%)			
			PW	PB	PE	PP
1	0.69	6-Hydroxyangelicin	0.40	—	—	—
2	0.89	Mannitol	—	0.43	—	—
3	0.91	3-Buten-2-one 1-(2,3,6-trimethyl phenyl)	1.37	0.23	—	—
4	0.96	Lactulose	—	0.28	—	—
5	0.97	Ectoine	—	1.81	—	—
6	0.98	Heptyl formate	0.45	0.38	—	—
7	0.99	Genipic acid	0.45	—	—	—
8	1.00	2,4-Diethylthiazole	—	1.56	—	—
9	1.05	2,5-Dioxopentanoate	2.18	—	—	—
10	1.24	2-Hydroxyethanesulfonate	0.40	0.39	—	—
11	1.27	2,6-Piperidinedicarboxylic acid	1.09	—	—	—
12	1.37	Pregabalin	1.99	—	—	—
13	1.38	N-acetylhistamine	1.46	—	—	—
14	1.38	Nipecotic acid	11.99	1.51	—	—
15	1.39	4-Guanidinobutanoic acid	—	0.25	—	—
16	1.44	Gln gly	1.87	—	—	—
17	1.52	Cytarabine	—	0.74	—	—
18	1.62	Ser val gly	—	2.46	—	—
19	1.74	Ormetoprim	0.29	—	—	—
20	2.02	6-Hydroxynicotinic acid	—	—	0.33	—
21	2.54	3,3,5-Triiodo-L-thyronine-beta-D-glucuronoside	—	0.35	—	—
22	3.13	2-Phenylacetamide	0.63	0.52	3.86	0.10
23	4.1	(S)-5′-deoxy-5'-(methylsulfinyl)adenosine	—	0.54	—	—
24	4.12	Adenine	—	1.88	—	—
25	4.12	Adenosine	0.39	14.99	0.47	—
26	4.19	Cordycepin	—	0.32	—	—
27	4.31	Patulin	0.42	—	—	—
28	4.34	4-Pyridoxic acid	—	0.27	0.36	—
29	4.35	Inosine	—	0.94	—	—
30	4.36	3′-Deoxyadenosine	—	—	0.41	—
31	4.39	*α*-[1-(ethylamino)ethyl]-p-hydroxy- benzyl alcohol	—	—	0.56	—
32	4.93	Selfotel	1.51	—	—	—
33	4.98	2-Acetyl-1-ethylpyrrole	3.72	0.30	5.22	0.08
34	4.99	3-Aminobenzamide	0.87	—	0.93	—
35	5.01	4-Hydroxydihydrocinnamaldehyde	—	—	0.36	—
36	5.11	Imidazoleacetic acid	—	—	0.79	—
37	5.16	Mequinol	0.34	2.55	—	—
38	5.36	2-Pyrocatechuic acid	0.62	—	0.85	—
39	5.36	L-tryptophan	1.52	0.44	—	—
40	5.39	Imidacloprid-guanidine	0.39	0.27	1.07	—
41	5.50	Dopamine	—	—	0.50	—
42	5.76	Methiocarb-sulfoxide	0.45	—	—	—
43	5.81	3-(4-methoxy-6-oxopyran-2-yl)butanoic acid	0.51	—	—	—
44	6.24	1-naphthylmethanol	—	—	0.89	—
45	6.41	1H-indole-3-carboxaldehyde	2.65	0.32	1.86	0.38
46	6.73	n-Acetylhomoproline	—	0.23	—	—
47	6.77	Methyl 3-methyl-1-butenyl disulfide	0.75	0.28	—	—
48	6.82	Aesculin	—	—	0.66	—
49	6.87	Oleic acid	—	—	0.47	—
50	6.89	Beta-carboline	—	—	0.86	—
51	6.98	3-Carboxypropyl trimethylammonium	0.43	—	0.39	—
52	6.99	Apigenin	—	—	8.48	11.28
53	7.28	trans-O-hydroxybenzylidenepyruvate	0.35	—	—	—
54	7.31	Eugenitol	0.85	0.22	0.84	—
55	7.54	Glycolic acid	—	—	—	0.18
56	7.59	2-Ketobutyric acid	—	—	—	0.12
57	7.61	Indoleacetic acid	0.44	—	0.43	—
58	7.62	4-Methyl-1-phenyl-2-pentanone	1.35	—	—	—
59	7.66	3-Methyl-1-phenyl-3-pentanol	8.05	—	0.72	—
60	7.69	3′-Methoxy-E,E-dienoestrol	0.36	—	0.70	—
61	7.79	2-Butyl-1-octanol	0.38	—	0.52	—
62	7.80	2-Hydroxy-8-methylchromene-2-carboxylate	0.43	—	1.91	—
63	7.84	Diflufenican	—	1.78	0.82	—
64	7.89	3-Methylbenzaldehyde	0.40	—	0.42	—
65	7.98	Luteolin 7-galactoside	6.59	2.42	0.76	—
66	7.99	2-Naphthol	2.30	0.24	0.94	—
67	8.16	Apiin	2.34	3.64	0.78	—
68	8.17	Ambolin	—	6.72	1.49	—
69	8.19	2-Hydroxymuconate semialdehyde	1.29	5.21	1.53	—
70	8.20	Decanoic acid, 3-amino-, (S)-	—	0.66	2.77	—
71	8.21	Indoleacrylic acid	0.54	—	1.82	—
72	8.22	Glycine	—	—	—	0.11
73	8.27	Aesculetin	0.11	0.56	4.32	13.28
74	8.27	Pelargonidin 3-O-glucoside	—	2.08	2.61	—
75	8.32	Peonidin-3-O-beta-galactopyranoside	0.18	1.95	2.97	—
76	8.46	2-Butyl-3-phenyl-2-propen-1-al	0.79	0.35	0.95	—
77	8.61	1,6-Dimethoxypyrene	0.30	—	2.08	—
78	8.65	Foliosidine	0.51	—	1.76	—
79	8.72	Ensulizole	2.23	—	1.53	—
80	8.73	3-Hydroxypropionic acid	—	—	—	2.61
81	8.84	Flavan skeleton	0.40	—	1.82	—
82	8.92	Chalconaringenin	—	4.87	—	—
83	9.01	Peonidin 3-(6″-acetylglucoside)	—	—	1.82	—
84	9.27	Mesylate	7.13	3.03	0.59	—
85	9.33	15-Methylpalmitate (2R,3R)-2-(3,4-dihydroxyphenyl)-3,5-	0.12	7.18	2.26	—
86	9.40	Dihydroxy-7-methoxy-2,3-dihydrochromen-4-one	1.86	1.17	—	—
87	9.70	*α*-Terpinene	0.45	0.51	0.37	0.47
88	9.80	4-Methyl-N-ethylcathinone	0.54	1.43	0.86	—
89	9.93	Perillyl acetate	0.65	0.26	1.39	—
90	10.27	5,6,7,8-Tetrahydro-2-naphthol	1.12	2.36	1.49	—
91	10.52	Cicaprost	—	0.25	2.47	—
92	13.72	Erucamide	2.27	0.75	0.80	0.09
93	13.91	Oxoproline	—	—	—	0.16
94	14.52	(2R,3S)-2-Hydroxy-3-isopropylbutanedioic acid	—	—	—	0.16
95	15.44	Vanillin	—	—	—	0.39
96	15.51	Lauric acid	—	—	—	0.17
97	15.80	DL-anabasine	—	—	—	1.79
98	15.93	Phthalic acid	—	—	—	0.35
99	16.01	Methyl jasmonate	—	—	—	1.44
100	16.23	D-arabitol	—	—	—	0.58
101	16.24	Ribitol	—	—	—	0.42
102	16.59	N-(2-hydroxyethyl)-iminodiacetic acid	—	—	—	0.10
103	16.68	Farnesal	—	—	—	19.37
104	16.72	S-carboxymethylcysteine	—	—	—	0.17
105	16.87	Gentisic acid	—	—	—	1.81
106	16.97	terephthalic acid	—	—	—	1.10
107	17.07	Farnesol	—	—	—	0.40
108	17.13	N-acetylisatin	—	—	—	0.16
109	17.64	Myristic acid	—	—	—	0.47
110	17.76	Fructose	0.78	—	—	—
111	18.08	Butylphthalide	—	—	—	18.42
112	18.09	Galactose	0.10	—	—	—
113	18.59	Coniferyl alcohol	—	—	—	0.82
114	18.70	1-Hexadecanol	—	—	—	0.12
115	19.45	4-Hydroxymethyl-3-methoxyphenoxyacetic acid	—	—	—	13.54
116	19.62	Palmitic acid	—	—	—	0.64
117	19.94	Myo-inositol	—	—	—	0.08
118	20.61	6-Hydroxy caproic acid dimer	—	—	—	0.35
119	21.17	Linoleic acid	—	—	—	0.78
120	21.40	Stearic acid	—	—	—	0.76
121	28.89	Cholestan-3beta-ol	—	—	—	0.25
122	30.62	Cholestane-3,5,6-triol, (3beta, 5alpha,6beta)-	—	—	—	0.82
		Total	84.35	81.88	75.86	94.32

RT^a^: retention time (min); Peak Area^b^: the average of the percentage of peak area relative to the total peak area (*n* = 3); “—“: not detected.

**Table 6:** Effects of monomeric compounds on the proliferation of HSC-LX2 cells (mean ± SD, *n* = 3).

Group	Dose (*μ*g·mL^−1^)	OD	Inhibition ratio (%)
Solvent control	—	0.164 ± 0.012	—

Blank control	0	1.210 ± 0.070	—

Model group(TGF-*β*1)	0.1	3.325 ± 0.138^##^	—

BJRG	60	0.939 ± 0.104^*∗∗*^	75.46 ± 2.11

Apigenin	15	2.746 ± 0.167^*∗*#^	18.33 ± 1.48
	30	1.816 ± 0.046^*∗∗*#^	47.72 ± 1.93
	60	1.071 ± 0.107^*∗∗*^	71.32 ± 2.41

5-Methoxypsoralen	15	3.337 ± 0.128	—
	30	3.382 ± 0.130	—
	60	3.454 ± 0.117	—

Apiin	15	3.238 ± 0.177^##^	2.73 ± 0.06
	30	2.716 ± 0.184^*∗*#^	19.26 ± 1.30
	60	2.349 ± 0.329^*∗*#^	30.87 ± 1.85

Rutin	15	3.412 ± 0.193	—
	30	3.201 ± 0.104^##^	3.92 ± 0.57
	60	2.825 ± 0.184^*∗*##^	15.81 ± 2.68

Kaempferol	15	3.462 ± 0.161	—
	30	3.186 ± 0.252^##^	4.38 ± 1.02
	60	2.629 ± 0.177^*∗*#^	22.02 ± 3.93

Luteolin	15	3.329 ± 0.202	—
	30	3.341 ± 0.175	—
	60	3.420 ± 0.330	—

Quercetin	15	3.186 ± 0.268^##^	4.39 ± 0.75
	30	2.337 ± 0.224^*∗*#^	31.26 ± 2.11
	60	1.798 ± 0.218^*∗∗*#^	48.28 ± 2.74

Mollugin	15	3.348 ± 0.152	—
	30	3.350 ± 0.183	—
	60	3.425 ± 0.159	—

Butylphthalide	15	3.029 ± 0.249^##^	9.36 ± 1.01
	30	2.366 ± 0.048^*∗*#^	30.33 ± 2.77
	60	1.367 ± 0.164^*∗∗*^	61.93 ± 3.51

Aesculetin	15	2.426 ± 0.245^*∗*#^	28.44 ± 4.01
	30	1.979 ± 0.122^*∗∗*#^	42.55 ± 3.14
	60	1.277 ± 0.273^*∗∗*^	64.90 ± 4.21

Compared with the blank control group (^#^*P* < 0.05, ^##^*P* < 0.01); compared with the model group (^*∗*^*P* < 0.05, ^*∗∗*^*P* < 0.01).

## Data Availability

The data used to support the findings of this study are available from the authors.
