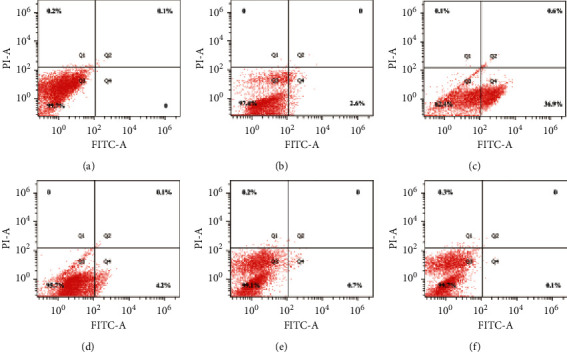# Corrigendum to “Antiliver Fibrosis Screening of Active Ingredients from *Apium graveolens* L. Seeds via GC-TOF-MS and UHPLC-MS/MS”

**DOI:** 10.1155/2022/9818920

**Published:** 2022-04-30

**Authors:** Ming Qiao, Jianhua Yang, Yao Zhao, Yi Zhu, Xiaomei Wang, Xinling Wang, Junping Hu

**Affiliations:** ^1^College of Pharmacy, Xinjiang Medical University, Urumqi 830011, China; ^2^Department of Pharmacy, The First Affiliated Hospital, Xinjiang Medical University, Urumqi 830011, China

In the article titled “Antiliver Fibrosis Screening of Active Ingredients from *Apium graveolens* L. Seeds via GC-TOF-MS and UHPLC-MS/MS” [1], concerns regarding duplicate images in Figure 1 were raised on PubPeer [2]. Specifically, Figures 1(c) and 1(d) appear to be identical. The authors explained that this duplication occurred due to an error introduced during the preparation of the manuscript and have provided a corrected image for Figure 1(c), shown as follows.

## Figures and Tables

**Figure 1:**